# Anetoderma Presenting as Neoplasms of Uncertain Behavior in an Elderly Female: A Case Report

**DOI:** 10.7759/cureus.97313

**Published:** 2025-11-20

**Authors:** Brian A Moreno, Moises Lutwak, Uros Rakita, Francisco Kerdel

**Affiliations:** 1 Dermatology, Lake Erie College of Osteopathic Medicine, Bradenton, USA; 2 Dermatology, Larkin Community Hospital, South Miami, USA; 3 Dermatology, Florida Academic Dermatology Center, Coral Gables, USA

**Keywords:** academic dermatology, clinical dermatology, dermatology, dermatology consult, dermatology outpatients, dermatology screening, general dermatology, inclusive dermatology training, investigative dermatology, medical dermatology

## Abstract

Anetoderma is a rare cutaneous disorder characterized by focal loss of dermal elastic tissue, often presenting as atrophic or herniated plaques. However, clinical presentations can vary, and atypical forms may resemble neoplastic or inflammatory lesions, complicating diagnosis. We present the case of a 78-year-old female who was evaluated for asymptomatic skin growths on the posterior neck, chest, and torso, reported to have been present for five years and slowly spreading. On physical examination, firm papules and subcutaneous nodules were noted on the left rib cage, left medial breast, and mid-trapezial neck. The lesions were initially diagnosed clinically as neoplasms of uncertain behavior, and two were biopsied using the punch method. Histopathology from the left rib cage revealed anetoderma, prompting reassurance and conservative management. At follow-up, anetoderma was also identified on the right medial superior chest. The patient remained asymptomatic, with no new lesions reported. This case highlights an uncommon presentation of anetoderma masquerading as subcutaneous neoplasms in an elderly patient with no known systemic disease or identifiable preceding dermatosis. It underscores the importance of histopathologic confirmation when evaluating lesions of uncertain behavior and expands the clinical spectrum of anetoderma, particularly in older adults. Given the lack of effective treatment options, accurate diagnosis is essential for guiding appropriate patient counseling and avoiding unnecessary interventions.

## Introduction

Anetoderma is an uncommon cutaneous disorder characterized by localized loss of dermal elastic fibers, resulting in skin that appears atrophic, wrinkled, or pouch-like on palpation and inspection [1]. Classically divided into primary and secondary forms, anetoderma may arise idiopathically or in association with a range of preceding dermatologic or systemic conditions. Although often described in younger adults, it can present across a broad age spectrum, and its clinical appearance may vary significantly. In certain cases, lesions may resemble benign or malignant neoplasms, leading to diagnostic uncertainty and the need for histopathologic confirmation. Because anetoderma can occasionally mimic benign or malignant neoplasms, especially when lesions present as papules or subcutaneous nodules, timely recognition is essential to avoid misdiagnosis. Fewer than several hundred cases have been described in the literature, and presentations in older adults remain uncommon.

Beyond its clinical appearance, several mechanistic pathways have been proposed to explain elastic fiber loss. Histologically, anetoderma is marked by a focal reduction or complete absence of elastic tissue in the dermis. Ultrastructural analyses have shown degeneration of elastic fibers, often accompanied by perivascular inflammatory infiltrates and fragmentation of the extracellular matrix, supporting theories of immune-mediated or enzymatic elastolysis [2]. Recent literature has also explored the potential role of prothrombotic states in its pathogenesis. Some patients demonstrate abnormalities such as antiphospholipid antibodies or deficiencies in protein C or protein S, implicating microvascular occlusion as a potential contributor to elastic fiber damage [3]. Anetoderma has even been reported in association with hematologic abnormalities and post-viral immune dysregulation, including cases arising after COVID-19 infection [4].

Several autoimmune diseases, most notably systemic lupus erythematosus (SLE), have been linked to anetoderma, with a proposed correlation between lesion development and the presence of antiphospholipid antibodies [5-7]. Cutaneous lymphoproliferative disorders, including B-cell lymphoma and marginal zone lymphoma, have also been reported as secondary causes [8,9]. Inflammatory dermatoses such as granuloma annulare may likewise precede the onset of anetoderma, supporting a reactive, post-inflammatory mechanism in some cases [10]. Lastly, familial and inherited forms, though exceedingly rare, have been described, further expanding the clinical spectrum of the disease [11].

This case report describes a 78-year-old female who presented with firm subcutaneous nodules initially suspected to be neoplastic in nature. Biopsy revealed anetoderma, despite the absence of typical clinical features or associated systemic disease. This case highlights an unusual presentation of anetoderma in an elderly patient and underscores the importance of biopsy in evaluating lesions of uncertain behavior.

## Case presentation

A 78-year-old female with no significant systemic symptoms presented to the dermatology clinic for evaluation of multiple asymptomatic skin growths on the posterior neck, chest, and torso. The lesions had been present for approximately five years with slow progression. On examination, firm papules and subcutaneous nodules were noted on the left rib cage, lower sternum, left medial breast in the 10-11 o'clock region, and mid-trapezial neck. The lesions were flesh-colored to slightly tan, non-tender, and exhibited intact overlying skin with no erythema or surface change. The patient denied pain, pruritus, or systemic complaints. No prior definitive diagnosis had been established, though she reported previous nonspecific biopsy results performed by an outside dermatologist.

**Figure 1:**
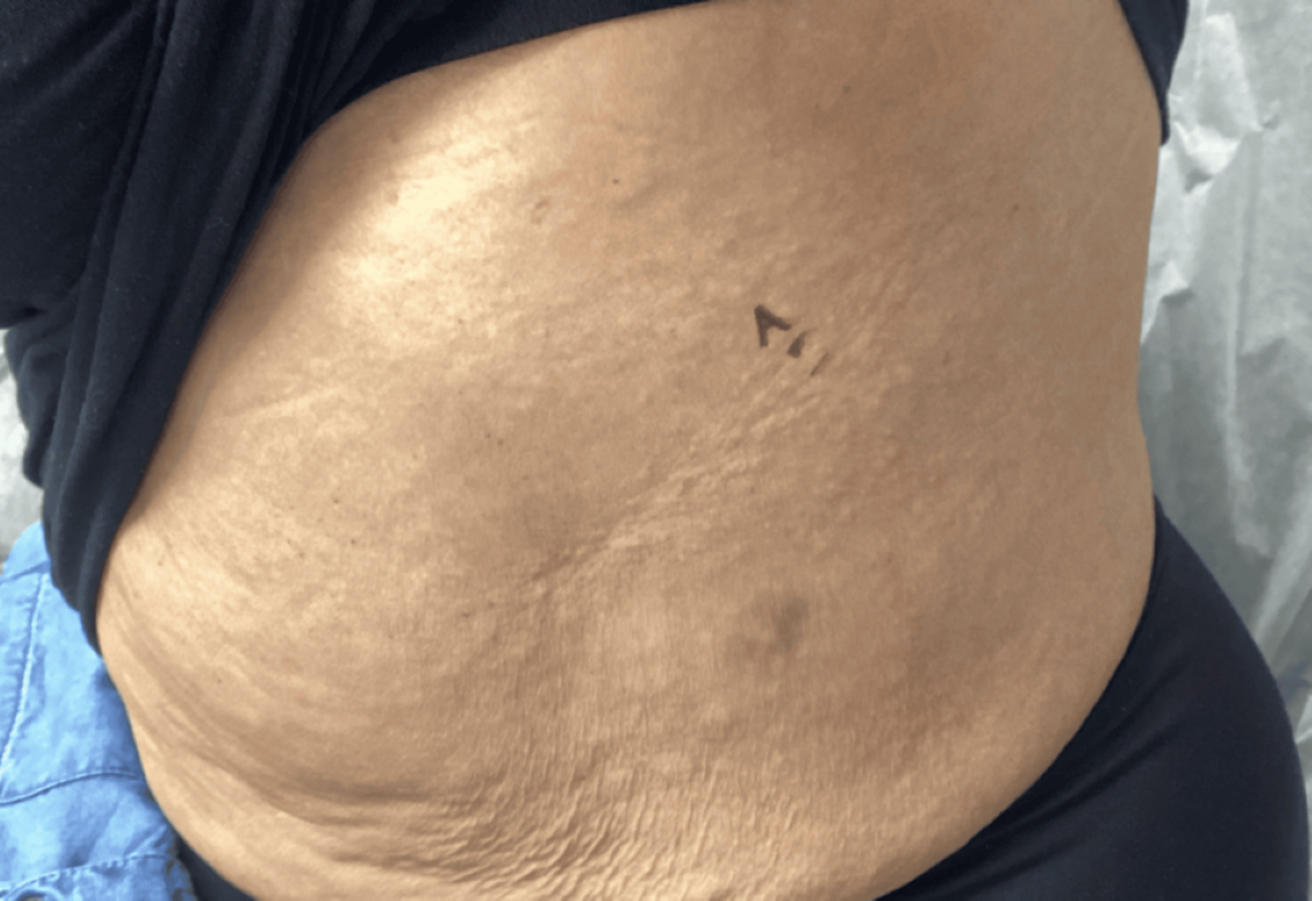
Clinical image of the left rib cage lesion before biopsy, later diagnosed histologically as anetoderma. The patient provided written consent for the use of clinical photographs; all images are fully de-identified. Lesion borders are visible without the need for arrows; no scale bar was required due to adequate anatomical context.

**Figure 2:**
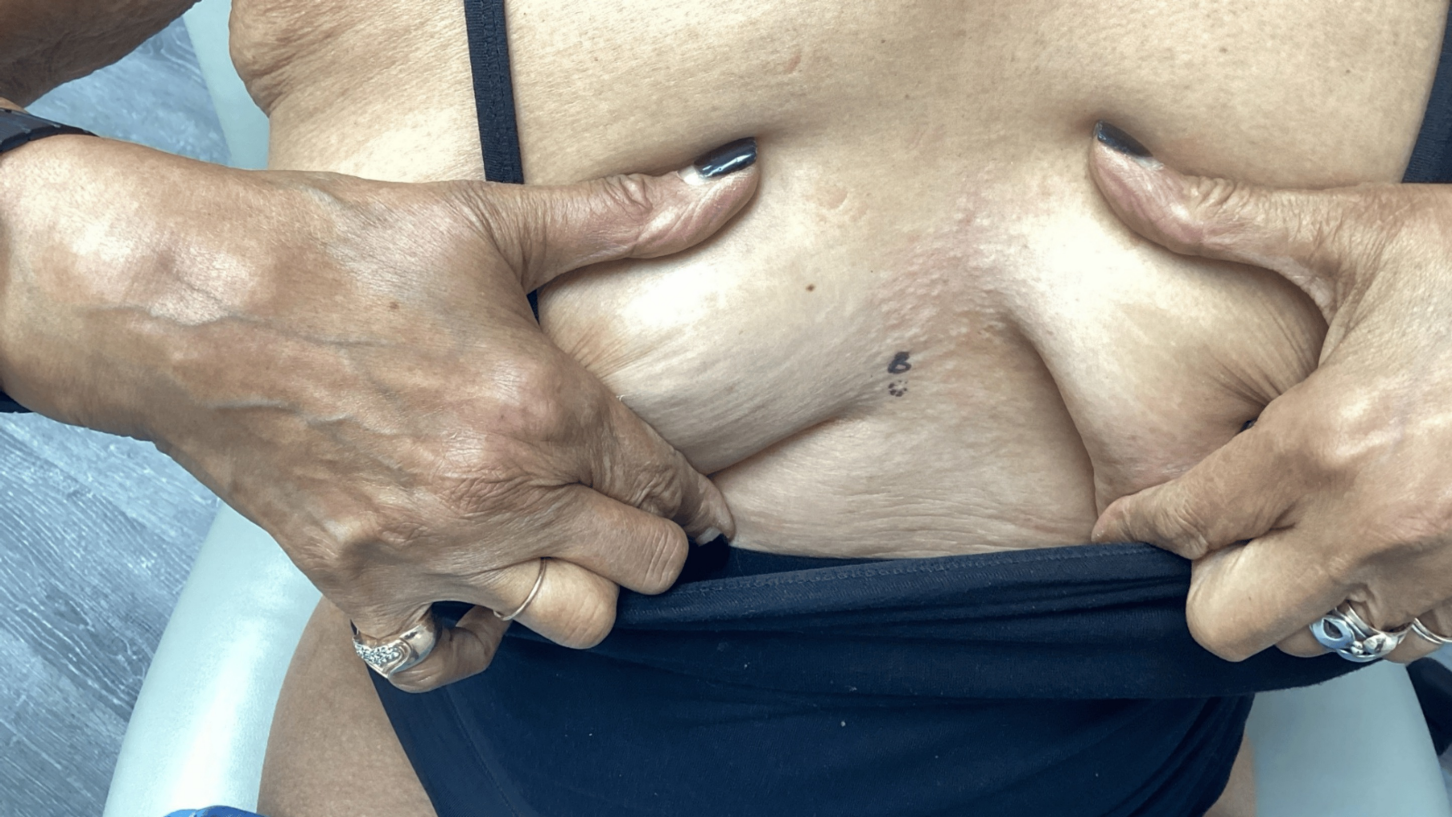
Clinical image of the lower sternum lesion before biopsy, later diagnosed histologically as anetoderma. The patient provided written consent for the use of clinical photographs; all images are fully de-identified. Lesion borders are visible without the need for arrows; no scale bar was required due to adequate anatomical context.

Given concern for neoplasms of uncertain behavior, punch biopsies were obtained from two lesions-one on the left rib cage and another on the lower sternum. Both biopsies were performed during the same clinical visit to evaluate lesions with similar morphology. Histopathologic evaluation of the left rib cage lesion revealed dermal changes consistent with anetoderma, characterized by a markedly decreased density of elastic fibers, occasional fragmented elastic fibers, and subtle perivascular lymphohistiocytic infiltrate with hemosiderin deposition and dermal telangiectasias (Figure 1). A corresponding Movat stain confirmed the loss of elastic tissue. The lower sternal lesion exhibited similar histologic findings (Figure 2). No cellular atypia, vasculitis, granulomatous inflammation, or malignancy was identified.

At a follow-up visit two months later, the patient remained asymptomatic and denied development of new lesions. Physical examination revealed additional soft, atrophic skin changes on the right medial superior chest, consistent with anetoderma. Based on the clinical and histopathologic findings, the diagnosis of anetoderma was confirmed, and the patient was reassured regarding its benign nature. No specific treatment was initiated, and she was counseled on the chronic course of the condition and instructed to return if new or concerning lesions developed.

## Discussion

Anetoderma is a rare disorder of elastic tissue characterized by localized loss of dermal elastin, leading to atrophic or herniated skin lesions. Traditionally, it presents as soft, wrinkled patches or sac-like protrusions and is most often observed in young adults, particularly females [1]. However, the clinical spectrum of anetoderma is broad and may deviate from classic textbook descriptions. In this case, the patient’s lesions appeared as firm, subcutaneous nodules, leading to a working diagnosis of neoplasms of uncertain behavior and prompting biopsy. This underscores the variability of clinical presentation and the importance of histopathologic confirmation, especially in elderly patients, where anetoderma is rarely considered early in the differential.

Histopathologic examination remains the gold standard for diagnosis. The defining feature is a marked reduction or absence of elastic fibers in the papillary and mid-reticular dermis. Ancillary staining, such as Movat pentachrome or Verhoeff-Van Gieson, can aid in visualizing elastic fiber loss. Electron microscopy further reveals fragmented, shortened, or absent elastic fibers along with occasional perivascular infiltrates and subtle dermal alterations [2]. These features support hypotheses that localized inflammation or enzymatic degradation may play a role in elastolysis.

Emerging literature also implicates systemic factors such as prothrombotic abnormalities. Several studies have demonstrated associations with elevated antiphospholipid antibodies, lupus anticoagulant, or deficiencies in anticoagulant proteins, suggesting that microvascular thrombosis may contribute to local ischemia and subsequent destruction of elastic fibers [3]. One recent case linked the onset of anetoderma to hematologic dysregulation in the setting of COVID-19, further supporting a multifactorial immune-thrombotic mechanism [4].

Autoimmune diseases, particularly systemic lupus erythematosus (SLE), represent another well-established association. In multiple reports, patients with SLE developed anetoderma in the context of antiphospholipid antibody positivity or anti-proliferating cell nuclear antigen (anti-PCNA) autoantibodies [5-7]. Though our patient did not display clinical or laboratory signs of autoimmunity, these connections are relevant when evaluating for secondary causes, especially when lesions are widespread or progressive.

Additionally, anetoderma may arise secondary to hematologic malignancies or lymphoproliferative conditions. B-cell lymphomas, including marginal zone lymphoma, have been implicated in rare cases [8,9]. In our patient, the presence of firm, subcutaneous nodules with no systemic symptoms raised concern for a lymphoid or neoplastic process, which was ultimately excluded by biopsy. This diagnostic ambiguity highlights the importance of maintaining a broad differential and using tissue diagnosis to prevent unnecessary interventions. Histologic evaluation excluded other elastolytic disorders such as mid-dermal elastolysis, as well as granulomatous or lymphoid processes, including lupus panniculitis and atrophic dermatofibroma.

Inflammatory dermatoses such as granuloma annulare have also been associated with secondary anetoderma [10]. These cases are typically preceded by more obvious cutaneous inflammation, which was absent in our patient. Finally, rare familial forms of anetoderma have been described, often presenting in childhood or adolescence with symmetric or generalized distribution. A report of two Japanese siblings with inherited anetoderma supports a potential genetic predisposition, although such presentations remain exceedingly rare [11]. A literature review identified very few reports of anetoderma presenting predominantly as firm subcutaneous nodules in elderly patients, and none describing a presentation closely mimicking neoplasms of uncertain behavior, suggesting this may represent an uncommon or possibly first documented variant.

In this case, the patient’s age, lack of preceding inflammation, and nodule-like presentation made the diagnosis challenging. Histology played a pivotal role in clarifying the etiology and guiding management. While there is no definitive treatment for anetoderma, establishing an accurate diagnosis allows for appropriate reassurance, monitoring, and avoidance of unnecessary procedures. This case expands the clinical spectrum of anetoderma and reinforces the need to consider it in the differential diagnosis of cutaneous nodules, particularly when initial clinical impressions suggest neoplasia.

## Conclusions

Anetoderma is a rare and often underrecognized dermatologic condition with a broad clinical spectrum. This case highlights an atypical presentation in an elderly female, where firm subcutaneous nodules mimicked neoplastic lesions, ultimately revealing anetoderma on histopathology. The absence of preceding inflammation, systemic disease, or classic atrophic morphology contributed to diagnostic uncertainty. This report underscores the importance of maintaining a broad differential when evaluating cutaneous growths of uncertain behavior and reinforces the value of biopsy in establishing a definitive diagnosis. Early recognition of anetoderma, even in atypical presentations, can prevent unnecessary interventions and allow for appropriate counseling and follow-up. Clinicians should consider anetoderma in the differential diagnosis of firm cutaneous nodules in older adults, particularly when initial impressions suggest neoplasia but histology reveals benign elastolysis.
